# Pedigree-Based Gene Mapping Supports Previous Loci and Reveals Novel Suggestive Loci in Specific Language Impairment

**DOI:** 10.1044/2020_JSLHR-20-00102

**Published:** 2020-11-13

**Authors:** Erin M. Andres, Kathleen Kelsey Earnest, Shelley D. Smith, Mabel L. Rice, Muhammad Hashim Raza

**Affiliations:** a Child Language Doctoral Program, University of Kansas, Lawrence; b Language Acquisition Studies Lab, University of Kansas, Lawrence; c Department of Neurological Sciences, University of Nebraska Medical Center, Omaha

## Abstract

**Purpose:**

Specific language impairment (SLI) is characterized by a delay in language acquisition despite a lack of other developmental delays or hearing loss. Genetics of SLI is poorly understood. The purpose of this study is to identify SLI genetic loci through family-based linkage mapping.

**Method:**

We performed genome-wide parametric linkage analysis in six families segregating with SLI. An age-appropriate standardized omnibus language measure was used to categorically define the SLI phenotype.

**Results:**

A suggestive linkage region replicated a previous region of interest with the highest logarithm of odds (LOD) score of 2.40 at 14q11.2-q13.3 in Family 489. A paternal parent-of-origin effect associated with SLI and language phenotypes on a nonsynonymous single nucleotide polymorphism (SNP) in *NOP9* (14q12) was reported previously. Linkage analysis identified a new SLI locus at 15q24.3-25.3 with the highest parametric LOD score of 3.06 in Family 315 under a recessive mode of inheritance. Suggestive evidence of linkage was also revealed at 4q31.23-q35.2 in Family 300, with the highest LOD score of 2.41. Genetic linkage was not identified in the other three families included in parametric linkage analysis.

**Conclusions:**

These results are the first to report genome-wide suggestive linkage with a total language standard score on an age-appropriate omnibus language measure across a wide age range. Our findings confirm previous reports of a language-associated locus on chromosome 14q, report new SLI loci, and validate the pedigree-based parametric linkage analysis approach to mapping genes for SLI.

**Supplemental Material:**

https://doi.org/10.23641/asha.13203218

Most children acquire language without any formal instruction. Contrary to common expectations, language does not come easily to all children. Specific language impairment (SLI) is a language disorder that delays the acquisition of language skills in children who have no hearing loss or other developmental delays (National Institute on Deafness and Other Communication Disorders, 2019). The estimated prevalence of SLI (excluding children with low/borderline normal nonverbal IQ) is 7% in the United States (Tomblin et al., 1997), an estimate that was recently replicated for children in England (Norbury et al., 2016). When children with borderline normal nonverbal IQ are included in these affected samples, they add approximately 3% to the percentage (Norbury et al., 2016). Language ability is associated with multiple aspects of quality of life including stronger social relationships and academic success (Conti-Ramsden et al., 2018; Johnson et al., 2010; Rice, 2017; Tomblin, 2014). The causes of language delay in children with SLI are not known.

Family aggregation and twin studies provide evidence that the language acquisition difficulties children with SLI experience are inherited (Bishop et al., 2006, 1995; Bishop & Hayiou-Thomas, 2008; Rice et al., 1998, 2020, 2018; Tallal et al., 2001). Twin studies of individuals with and without SLI have revealed strong heritability estimates for multiple speech and language phenotypes in dizygotic and monozygotic twins (Bishop & Hayiou-Thomas, 2008; Rice et al., 2020, 2018). Heritability estimates vary, ranging from .20 to .94, depending on the phenotype, age of children, and dimensions of language assessed. Of interest here, standardized omnibus language measures (that assess multiple domains of language) are often in the higher heritability range (Bishop & Hayiou-Thomas, 2008; Rice et al., 2020, 2018).

Although SLI is known to aggregate in families (Rice et al., 1998; Tallal et al., 2001), family-based genetic studies in SLI pose challenges. SLI is a complex disorder, with age-variable language outcomes among individuals with SLI (although with similar overall developmental outcomes), that does not seem to align with a simple Mendelian inheritance pattern (Strachan et al., 2014; van Heyningen & Yeyati, 2004). Mendelian disorders follow Mendelian laws of inheritance characterized by the involvement of factors in a single gene, which can be heritable (changes in germ cells) or not heritable (de novo/new changes). Classification of Mendelian traits is dichotomous, while complex traits are those that affect individuals on more of a continuous spectrum of phenotypes (Strachan et al., 2014). Under simple Mendelian inheritance, contributing gene mutations show a complete genotype–phenotype correlation or complete disease penetrance (Jordan, 2014). Non-Mendelian traits are more likely the result of mutations in a single gene or multiple genes transmitted from parents to offspring under an undefined inheritance pattern with variable disease penetrance (van Heyningen & Yeyati, 2004). However, the expression of such disorders can be caused by a combination of genetic and environmental factors, further complicating the discovery of contributing factors in complex disorders, especially those that are phenotyped behaviorally, like SLI (Jordan, 2014; van Heyningen & Yeyati, 2004).

Linkage analysis is often the first step in family-based genetic investigation to narrow regions to identify causative genes and variations. The purpose of parametric linkage analysis is to identify coinheritance of chromosomal loci with a phenotype (Read & Strachan, 2011). Linkage is estimated by logarithm of odds (LOD) scores. In parametric linkage, factors such as mode of inheritance, disease frequency, and disease penetrance must be set to complete the analysis. The mode of inheritance can be set to dominant or recessive with variable disease penetrance. Although the use of multiple models during analysis is not ideal, past studies have shown it produces powerful results for analysis of complex phenotypes (Abreu et al., 1999; Bartlett et al., 2002; Greenberg et al., 1998). Traditional LOD score-based gene mapping methods are conventionally successful for simple Mendelian disorders, but similar approaches have also been successful for complex genetic disorders in which the mode of inheritance is not determined (Abreu et al., 1999). Linkage analysis methods utilizing dominant and recessive inheritance models were tested against a nonparametric linkage model and shown to be more powerful for heterogenous phenotypes (Abreu et al., 1999). Pedigree-based analysis of complex disorders with a well-defined phenotype serves to reduce trait variance across related individuals (Neale & Cardon, 2013). At the same time, the power of linkage in complex disorders is influenced by variable disease penetrance, phenocopy rate, and unknown segregation patterns (Clerget-Darpoux & Elston, 2007; Ott et al., 2011).

Multiple genetic studies of SLI are reported, with mixed outcomes for linkage and association; consistently high heritability estimates and genetic loci are reported (Reader et al., 2014; Rice et al., 2020; Smith, 2007). SLI is heterogeneous genetically and phenotypically (Bishop & Hayiou-Thomas, 2008). The SLI Consortium (SLIC) completed the first linkage analysis in individuals with SLI, using small nuclear families and analyses relying on sib-pair information (SLIC, 2002). Genetic analyses in nuclear SLI families were completed for three phenotype measures: (a) a test of nonword repetition (NWR) measuring phonological short-term memory (Gathercole et al., 1994), and omnibus standard scores on the (b) Receptive and (c) Expressive Language subtests of the Clinical Evaluation of Language Fundamentals–Revised (CELF-R; Semel et al., 1987). The resulting significant loci were reported on chromosome 16q24, linked with the NWR test, and on chromosome 19q13, linked with the CELF-R Expressive Language subtest (SLIC, 2002). However, in the follow-up targeted genetic study including new families, both linkage regions (chromosome 16q and 19q) were linked to the NWR test, whereas no linkage was observed with the CELF-R Receptive or Expressive subtests, counter to the previous findings (SLIC, 2004).

Similarly, an investigation of Canadian families phenotyped as reading impaired (RI), language impaired, or clinically impaired showed linkage on chromosome 13q; subsequent follow-up investigation of American families replicated the linkage on 13q (Bartlett et al., 2004, 2002). Participants in this study were grouped based on their performance on age-appropriate standardized measures of language and non-verbal intelligence, a battery of other tests, and a family history interview (Bartlett et al., 2004, 2002). The most significant linkage was revealed at 13q21 with an LOD score of 3.92 for individuals identified with RI (Bartlett et al., 2002). It was hypothesized that the RI group showed a reading deficit as a result of underlying language impairment (Bartlett et al., 2002). When an additional 22 American families were included in the analysis, an LOD score > 4.0 was noted at two locations on chromosome 13q (Bartlett et al., 2004). More recently, significant linkage overlapping with the identified region on 13q was mapped in an extended family with a history of verbal trait disorder (Truong et al., 2016). These studies provide support for a pedigree focused approach in the study of SLI, especially with appropriate phenotype measures.

Founder populations and consanguineous families provide a unique opportunity for family-based analysis. The founder-based population of Robinson Crusoe Island, near Chile, has a high incidence (35%) of SLI (Villanueva et al., 2011, 2008). Research participants, who lived on Robinson Crusoe Island, were comprehensively assessed with a nonverbal IQ measure, multiple language measures (including omnibus standard scores) in Spanish, and screened for hearing impairments, motor difficulties, and comorbid diagnoses (Villanueva et al., 2010, 2011). Family-wide parametric linkage analysis under autosomal dominant and recessive models of inheritance did not show significant linkage (Villanueva et al., 2011). However, nonparametric linkage analysis resulted in five significant linkage loci, including chromosome 6q, 7q, 12, 13, and 17, suggesting a polygenic pattern of SLI transmission (Villanueva et al., 2011). Similarly, linkage mapping and homozygosity mapping of extended consanguineous families, with language deficits, ascertained from Pakistan and phenotyped with a vocabulary measure, resulted in multiple suggestive loci: chromosome 2q, 5p, 8q, 14q, 17q, and 22q, suggesting locus heterogeneity (Andres et al., 2019).

Another approach considered regions previously linked to the phenotypically related impairment of reading disorder (RD). Previous behavioral investigations support a hypothesized relationship between SLI and RD, such that early language impairments are predicative of later reading impairments (Adlof & Hogan, 2019; Catts et al., 2006, 2005; Snowling et al., 2000). An earlier genetics study of SLI using a cohort of individuals and family members with SLI (*N* = 322), compiled at the University of Kansas (KU) Cohort, targeted the chromosomal loci 1p36, 3p12-q13, 6p22, and 15q21 (Rice et al., 2009). Sib-pair and family-based analyses utilized the full sample at targeted loci (Rice et al., 2009). Linkage analysis was performed using quantitative and categorical phenotypes. Nonparametric linkage analysis was performed in MERLIN for the categorical phenotypes. LOD scores above 0.6 were found for the standardized omnibus language categorical and quantitative phenotypes within the chromosome 1p36, 6p22, 7q31, and 15q21 loci (Rice et al., 2009). This targeted approach could be applied to novel populations, especially when the phenotype measures overlap. This indicates the possibility of an overlap in reading and language phenotypes, both genetically and behaviorally. Also, of note, several individuals included in the study of targeted RD loci are members of the families included in the current genome-wide linkage mapping study.

The power of family-based methods of genetic studies of SLI is supported by early linkage studies and more recent studies utilizing unique populations and exome sequencing (Andres et al., 2019; Rakhlin et al., 2013; Truong et al., 2016; Villanueva et al., 2011, 2015; Wiszniewski et al., 2013). Such studies have provided strong evidence for candidate genes including *TM4SF20*, *NFXL1*, and *SETBP1* (Rakhlin et al., 2013; Villanueva et al., 2015; Wiszniewski et al., 2013). One study of note is the family-based Robinson Crusoe Island investigation of a founder population, which underscores the need for further family-based study (Mountford et al., 2019; Villanueva et al., 2011, 2015). Genetic studies in the founder population identified a protein coding change in *NFXL1* with a higher frequency in individuals from the population with language impairment (Villanueva et al., 2015). Upon sequencing of *NFXL1* in SLIC probands, three more protein coding variations were identified in four additional SLI cases (Villanueva et al., 2015). *NFXL1* only explains a fraction of SLI cases, although with large effect (Villanueva et al., 2015). The combination of initial behavioral investigation (indicating a high rate of language impairment in the population), family-based linkage, and homozygosity mapping ultimately contributed to the utility of next-generation sequencing (NGS) and convincing exome sequencing results (Villanueva et al., 2011).

Across the reported linkage regions, many candidate genes have been identified and these studies indicate that more genes are involved in SLI than previously expected (Chen et al., 2017; Mountford & Newbury, 2018; Mountford et al., 2019). The current list of language impairment candidate genes is extensive, although across studies and reviews about 30 candidates are more consistently and recently discussed (Chen et al., 2017; Mountford & Newbury, 2018; Mountford et al., 2019). These reports are crucial for further investigation to acknowledge in analyses and reporting.

Our study uses the logic of the previous investigations to perform familywise genetic analyses of our sample. The goal of our study is to map loci, which can be used to identify candidate genes and rare variations of large effect associated with SLI in our sample and across previous and future studies in the field. The current study analyzed probands with SLI and their family members, assessed longitudinally (e.g., Rice & Hoffman, 2015). The rate of SLI in the family members (of the KU cohort) is significantly higher than the rate of SLI and related phenotypes in members of control probands (Rice et al., 1998). The elevated rate of SLI in family members is reflected in the six families investigated in the current study. We used traditional family-based LOD score methods to map genetic loci to performance on age-appropriate standardized omnibus language assessments. The rationale for this approach is to reduce trait variance within complex phenotypes at the behavioral level and capture the genetic transmission.

## Method

### Participants and Ethics Approval

**Figure 1.**
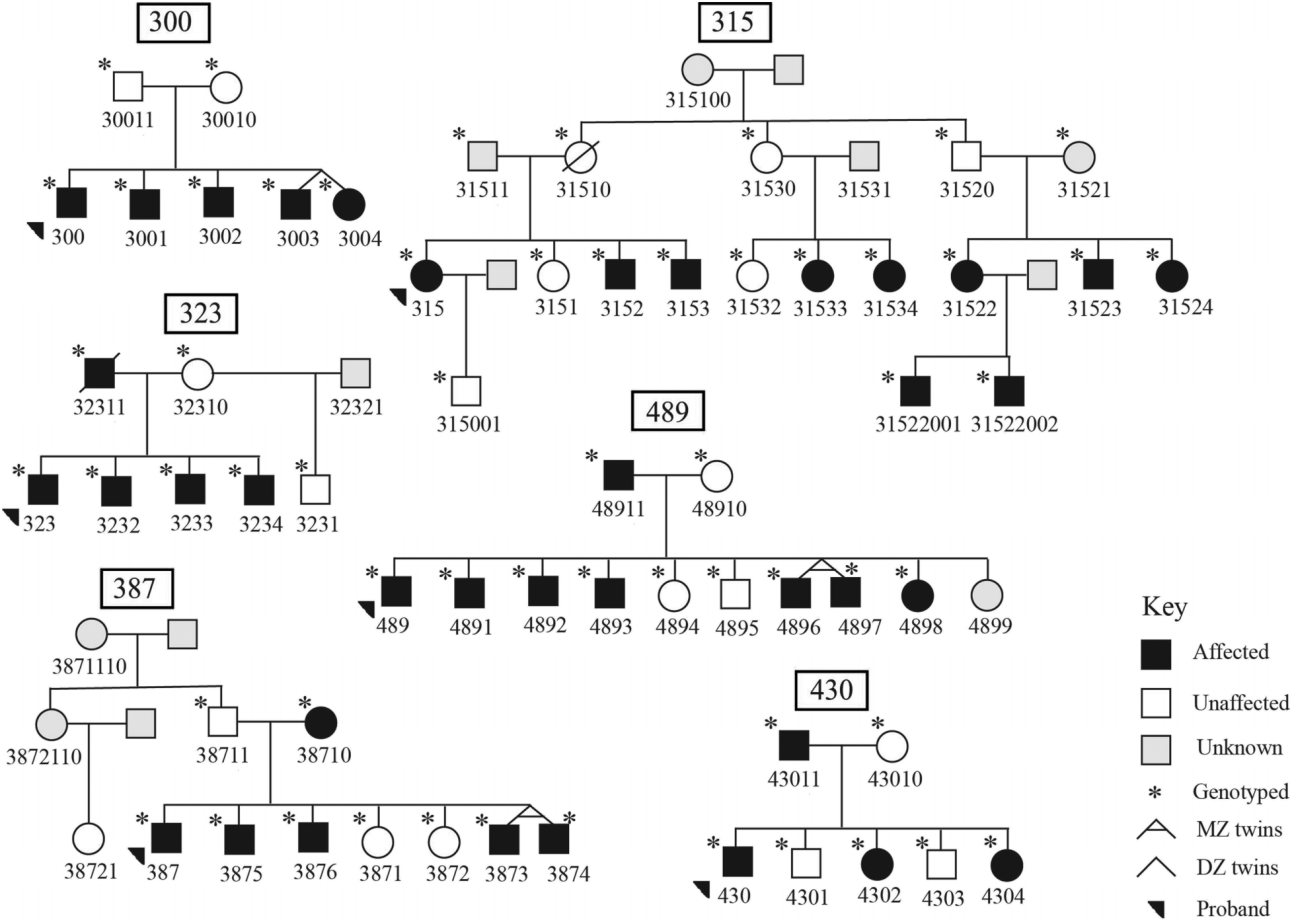
Six pedigrees used for linkage mapping. Family 315 Branch 1 (proband branch) includes descendants of 31510 and 31511, Family 315 Branch 2 includes descendants of 31520 and 31521, and Family 315 Branch 3 includes descendants of 31530 and 31531.

The families included in this study are a part of a larger ongoing longitudinal study in Dr. Mabel Rice's Language Acquisition Studies Lab at the KU. Most of the families were recruited from school speech pathology caseloads, except Family 315, who self-referred to the study. The institutional review board at the KU approved the study (Institutional Review Board No. 8223) and we acquired appropriate informed consent from all participants. Parents provided consent for all participants under 18 years of age. In total, 60 participants make up the six families, including six probands, 12 parents, 28 siblings, and 14 other relatives; 38 individuals are categorized as affected (see Figure 1). Affection status was determined based on standard scores from age-appropriate standardized omnibus language measures (described in detail below). As noted in the opening review of precedents in the literature, omnibus standard scores provide the same psychometric index across a wide age range, which is needed in family studies, and these measures have shown sensitivity to genetic effects in previous studies. The families in the current analysis were chosen based on their size, the high proportion of affected individuals, and availability of extended family members. Twin pairs are present in three families, one dizygotic (Family 300) and two monozygotic twin pairs (Families 387 and 489; see Figure 1). All six families are Caucasian.

Family 315 is the largest in the study; 18 individuals were available for single nucleotide polymorphism (SNP) genotyping and behavioral data was collected from 16 individuals. Genetic and language data were collected in Family 315 from three nuclear families (branches) descending from 315100. Branch 1 refers to the proband branch (descendants of 31510 and 31511), Branch 2 refers to the descendants of 31520 and 31521, and Branch 3 refers to the descendants of 31530 and 31531. Importantly, since the completion of genotyping in this study, additional behavioral information was collected in family members of Branch 2, including that one individual has a diagnosis of a rare syndromic condition, which presents with multiple severe phenotypes (not shown in Figure 1). This individual was not included in the present analysis, as their genetic and behavioral data were not available at the time of the genotyping. We note the existence of the rare condition in a young member of the family as of possible import for interpretation of possible outcomes, which we return to in the Discussion section.

### Phenotype Measures

A full list of the phenotype measures collected as part of the ongoing longitudinal study of families in Rice's lab is described by Rice et al. in an earlier publication (Rice et al., 2009). Probands entered the study as children affected with SLI. The exclusionary criteria were met by four entrance screening criteria: no cognitive impairment (average or above average nonverbal IQ), no hearing loss, no other diagnoses of developmental delay, neurological disorder or autism at the initial time of assessment (based on parent report), and intelligible speech. There were no probands with childhood apraxia of speech (CAS; https://www.asha.org/public/speech/disorders/childhood-apraxia-of-speech/), an uncommon motor speech disorder that makes it hard to speak, nor are there any in the full sample of participants. In addition, probands were monolingual native speakers of English and screened for nonstandard dialects. Family members (parents and siblings) of probands were also monolingual native speakers of English, without diagnosis of neurological conditions related to language, without hearing loss, and ≤ 55 years of age. Family members were not screened/exempt from participation for cognitive impairments or other developmental delays or autism at the initial time of assessment (based on parent report). Intelligible speech was required for validity of some of the phenotypes. Persons with hearing loss were not assessed/included in the study because the phenotype measures are not valid estimates of their language abilities.

Speech articulation was assessed with the Goldman-Fristoe Test of Articulation–Second Edition (Goldman & Fristoe, 2015). All participants were also assessed with an age-appropriate standardized nonverbal IQ assessment: Columbia Mental Maturity Scale for children between age 3;6 and 6;11 (years;months), Wechsler Intelligence Test for Children–Third Edition for ages 7 years through 16 years, and the Wechsler Intelligence Test for Adults–Third Edition for ages 17 years and older (Burgemeister et al., 1972; Wechsler, 1991, 1997). Participants recruited as children (i.e., probands, siblings, and young extended family members) were followed longitudinally, with annual administration of standardized measures. Parents, aunts, uncles, and other older extended family members were tested 1 time.

### Omnibus Language Measure

The omnibus language scores were obtained from a standardized language measure appropriate for the participant's age. Standardized omnibus language measures are designed to assess multiple dimensions of language, and age-standardized versions have been developed across a wide age range, following psychometric standards for reliability and validity. Although they do not measure all possible aspects of language acquisition, they are generally robust for broadly defined semantic and syntactic dimensions of language, across expressive versus receptive task demands. The omnibus language measures were chosen based on their high levels of reliability and validity, which is verified by monitoring of test–retest reliability across times of measurement in this longitudinal study. We note any potential psychometric weaknesses work against our hypotheses of genetic risk in the families.

The current study began following participants longitudinally in 1993 using an accelerated longitudinal design in which multiple age cohorts are sampled, such that children vary continuously in age at first assessment. The measures and their versions have remained the same throughout the course of the study in order to avoid confounds related to different items sets and different norming samples. It is essential to minimize this type of possible measurement error when comparing performance over time both within and across individuals. Training in the administration of the tasks includes reading the manuals, practice administration with adult trainers in the lab, observed administration with practice children in the lab, and followed by observed administration in the field with actual participants before being authorized to administer assessments independently. Routine intermittent live observations of actual data collection are conducted for monitoring reliability and validity of task administration, supplemented by viewing of video recordings of each session. Scoring of outcomes is also monitored and checked at various levels of processing, involving the examiners and other team members, on an ongoing basis. The measures were versions of two tests available at the outset of the study: for ages 4–6;11, the TOLD-2–Primary Spoken Language Standard Score (Newcomer & Hammill, 1988), and for 7 years of age through adulthood, the CELF-3 (Semel et al., 1995). Age norms on the CELF-3 stop at age 21;11. Our age range for this measure is 6.03–43.43 years. Thus, standard language scores for older adults in this study were based on norms for age 21;11, based on the assumption that language skills do not vary much per person, relative to the outcomes at 22 years, for the tasks included in the CELF-3.

We explored this expectation in our database, which includes participants with longitudinal assessments from childhood into adulthood. The sample was annually assessed beyond the ceiling age level. They entered as SLI (*N* = 18) or Controls (*N* = 10). The oldest time of sequential annual assessments was for one participant in the SLI group, a total of 13 assessments with the final at 33.69 years; for the Control group, the oldest was 30.11, a total of 12 assessments. For a comparison between “early adulthood” and “adulthood,” we calculated average omnibus standard scores per person in the age range 18–21 (“early adulthood”) and 22+ years (“adulthood”). For the SLI group, the difference between these averages ranges from 2 to 6 standard score points (within standard error of measurement) for all but one participant who had a traumatic experience that coincided with two lower scores that bounced back into the usual level in subsequent assessments. For the Control group, the range in differences for the “early” average versus the “later” average is 2–7, again within standard error of measurement, with one exceptional case of a period of three consecutive lower-than-average scores that later resolved to the previous levels. Overall, in our data, the general pattern of outcomes met our assumption that standard scores on the CELF-3 were unlikely to increase with age. Furthermore, we note that if language skills do improve in adulthood, then the use of younger age norms would inflate the adult standard scores, resulting in a lower likelihood of affection status, which would work against the study hypotheses. If such an inflation did occur, it is very small or nonexistent, as the average omnibus language standard score among proband parents in this study is 88.55 (*SD* = 15.16). Finally, recent analysis in this sample shows high test–retest reliability, with correlations between occasions of measurement ranging from *r* = .715 (*p* < .0001) to *r* = .975 (*p* < .0001), offering further support for stability of measurement over time.

Individuals with a standard score ≤ 85 on an age-appropriate standardized omnibus language measure were categorized as affected. Following precedents in previous longitudinal studies for assigning affectedness status, individuals were classified as affected based on their lowest standardized omnibus language score across all occasions of measurement. This method is consistent with those used in the SLIC studies where past or current language performance was used to identify probands (Falcaro et al., 2008). Importantly, the lowest performance estimate also captures the affectedness status of siblings. Individuals who never scored in the affected range across times of measurement (i.e., standard score > 85) were classified as unaffected, and those who did not complete a standardized omnibus language measure were assigned an unknown status. The age range for participants was 4.01–43.43 years; standard score range was 50–118 (lowest is a proband, and highest is a parent). Overall, on the phenotype level, the individuals seemed to share the common description of SLI. Note that the probands were recruited with exclusionary criteria to rule out possible confounding conditions. Siblings and parents could be affected as SLI, or with developmental disorders including autism. One sibling with autism is included among the participants in this study. The incidence of other conditions, such as autism or other developmental disorders, in our full database seems to align with population estimates. In this study, the family members, including the probands, were generally undetectable in a general population. Recent studies document that children with SLI are unlikely to be detected by their classroom teachers (Chrisopulos & Kean, 2020). Parents often comment that, if they scored low on testing (results are provided upon request, but few make the request), they were just like their child when they (a parent) were little (indicating one of the children who scored as affected, although that information was known only when parents asked). Almost all the parents were employed; some had advanced degrees, and others had high school degrees; employment included high-level engineering design, management positions, private businesses, and service positions.

### Genetic Analyses

#### DNA Collection and Preparation

Saliva was collected from participants at the time of initial or follow-up behavioral assessment. Saliva samples/buccal swabs were collected using the Oragene-Discover OGR-500 or OGR-575 Kits (DNA Genotek, Oragene). DNA was purified from the saliva samples using a standardized protocol with the Oragene prepIT⸰L2P kit. Extracted DNA was resuspended and stored in 1x Tris-EDTA (1X TE) buffer at −80°C for long-term storage.

#### Linkage Simulation

To obtain estimates of linkage, we performed familywise 2-point parametric linkage simulations using the FastSLink package in easyLINKAGE-Plus (Hoffmann & Lindner, 2005). Linkage simulation was tested in each family under dominant and recessive modes of inheritance with complete penetrance, consistent with our planned linkage analyses. Simulation used 1,000 replicates of one marker at a time, across the autosomal genome, with four equally frequent alleles and a 0% phenocopy rate with a 0.05 increment of recombination fraction. The primary goal of the linkage simulation was to determine the familywise maximum estimated LOD (ELOD) scores across the whole genome.

Suggestive and significant LOD score thresholds for complex traits have been proposed based on previous linkage simulations (Lander & Kruglyak, 1995). Proposed suggestive LOD score thresholds range between 1.9 and 2.4, while significant LOD score thresholds range between 3.3 and 3.8, depending on the family relationships tested (Lander & Kruglyak, 1995). In the current study, suggestive regions were defined around SNPs with calculated LOD scores greater than 1.9, consistent with suggestive linkage thresholds. Additional regions were defined around SNPs with calculated LOD scores greater than 1.2, in the case that they overlapped with regions previously linked to SLI or related phenotypes (see Supplemental Table S1). Related phenotypes included in the search were performance on NWR tests, autism spectrum disorders (ASDs), CAS, and RD (see Supplemental Table S1). The boundaries for all reported regions were determined based on the segment in which the majority of the markers surrounding the highest LOD score showed a positive LOD score.

#### SNP Genotyping

SNP genotyping was performed on the Illumina Infinium QC Array-24 that has 15,949 SNP markers equally spaced throughout the genome (https://www.illumina.com/products/by-type/microarray-kits/infinium-qc.html). All DNA samples were diluted with 1X TE buffer to 50-ng/ul concentration, and 200 ng of DNA was used for SNP genotyping. Genome-wide SNP genotyping was outsourced to the Genetic Resources Core Facility at Johns Hopkins University School of Medicine. Genotype data were consistent with reported family relationships in all individuals (call rate above 99.8%). The array has 12,032 SNPs distributed across the autosomal chromosomes, though 38 were located within pseudo-autosomal regions. Additionally, 3,917 SNPs were located on the sex chromosomes (X chromosome = 1,840 markers; Y chromosome = 1,401 markers; XY chromosome = 535 markers) and mitochondrial DNA (141 markers).

#### Parametric Linkage Analysis

Whole genome parametric linkage analysis was used to identify candidate chromosomal loci in the six families. Linkage analysis was performed using Superlink-Online SNP 1.1, a publicly available genetic linkage analysis program at http://cbl-hapw.cs.technion.ac.il/superlink-snp/ (Silberstein et al., 2012). Superlink-Online SNP 1.1 has a graphical interphase, allowing it to handle large pedigrees and thousands of SNP markers for single-point linkage analysis.

Linkage analysis was performed in each family individually using autosomal dominant and recessive inheritance models with variable disease penetrance. Previous studies recommended using reduced disease penetrance levels when working with complex phenotypes (Hodge et al., 1997). For each inheritance model, we performed analysis at two disease penetrance levels, complete (99%) and reduced (70%; see Table 1). A rare disease allele frequency of 0.001 with a 0% phenocopy rate was used in all analyses.

**Table 1.** Penetrance levels used for analyses.

Inheritance pattern	Disease penetrance	WT/WT homozygous	WT/MUT heterozygous	MUT/MUT homozygous
Dominant	Complete	0.00	0.99	0.99
	Reduced	0.00	0.70	0.70
Recessive	Complete	0.00	0.00	0.99
	Reduced	0.00	0.00	0.70

*Note.* WT = wild type; MUT = mutant.

Out of 15,949 SNPs, a total of 13,834 SNPs (autosomal and X chromosome) were used in the Superlink-Online SNP 1.1 analysis. Superlink-Online SNP 1.1 has a cleaning tool, which removes markers that are uninformative or erroneous for various reasons (Silberstein et al., 2012). In total, the cleaning tool removed 3,983 markers (includes 1,773 X chromosome markers) across the six families, leaving 9,851 informative markers for linkage analyses.

The linkage program MERLIN was used to draw haplotypes in Family 315 (Abecasis et al., 2002). Given the family history of rare syndromic conditions in one part of Family 315 (Branch 2), additional analyses were performed, including analysis of the individual branches and analysis removing one branch at a time. Loss of heterozygosity analysis was performed in Family 315 using Homozygosity Mapper (http://www.homozygositymapper.org; Seelow et al., 2009).

## Results

Six probands with SLI and their family members were included in genome-wide parametric linkage analysis, and the highest LOD scores were compared with the maximum ELODs (see Table 2). In alignment with the simulated results, we report suggestive evidence of linkage to three independent loci in three families (300, 315, 489; see Table 3), while evidence of suggestive or significant linkage was not identified in the other three families (323, 387, and 430; see Figure 1 and Supplemental Figure S1). In Family 387, under the recessive model of inheritance, two markers spanning chrX:89712929-90955058 showed single-point LOD scores equal to the maximum ELOD (1.81; see Table 2). This agrees with visual inspection of the mode of inheritance in Family 387, which shows the possibility of sex-linked recessive inheritance (see Figure 1).

**Table 2.** Maximum estimated LOD scores in all families.

Family	Inheritance pattern	
	Dominant	Recessive
300	NA	2.41
315	4.09	4.63
323	0.87	0.90
387	1.81	1.81
430	1.20	1.20
489	2.41	2.41

*Note.* We did not perform simulation under the dominant model of inheritance in Family 300 because both parents are unaffected for the omnibus phenotype while all children are affected. NA = not available.

**Table 3.** Suggestive specific language impairment loci (LOD score > 2.2).

Family	Locus	hg19 position (Mbp)	rsIDs	Single-point LOD scores		No. of genes
				Dominant inheritance	Recessive inheritance	
300	4q31.3–q34.3	154.88–179.98	rs1878449–rs12499500	1.20	2.41^a^	106
315	15q23–q25.2	68.68–83.22	rs1075991–rs1267657	1.46	3.06	196
489	14q11.2–q13.3	20.83–37.06	rs2700–rs1955739	1.50	2.40^a^	216

*Note.* Mbp = megabases.

a LOD scores that reached the maximum estimated LOD.

The highest calculated single-point LOD score was greater than 1.9 in the reported suggestive loci and matched the maximum ELOD in Family 300 and 489 (see Table 3). The highest single-point and multipoint LOD score of 2.4 was obtained in Family 300 to 4q (154.9-180.0 Mbp) and in Family 489 to 14q (20.82–37.06 Mbp), under the recessive mode of inheritance (see Table 3). However, in Family 315, the highest single-point LOD score of 3.06 to rs11072823 at 15q (79.29 Mbp) was less than the maximum ELOD score (see Table 3). Genes within the suggestive regions are listed in Supplemental Table S2 (4q), Supplemental Table S3 (15q), and Supplemental Table S4 (14q). To provide additional context for the suggestive regions, the background linkage and the families with no significant linkage, the genome-wide linkage results for all six families are presented in Supplemental Figure S1. There were 10 additional markers in Family 315 with LOD scores greater than 2.0 (see Supplemental Figure S1 and Supplemental Table S5). However, the markers, in the vicinity of the highest scoring markers, showed negative LOD scores (see Supplemental Figure S1).

The reduced single-point LOD score (compared to the maximum ELOD) in Family 315 led us to perform branch-wise linkage analysis, especially considering the presence of a rare syndromic condition (details of diagnosis not available) in Branch 2. We performed linkage analysis separately in Branch 1, Branch 2, and Branch 3 and all possible combinations of these branches (see Figure 1 and Supplemental Figure S2). The highest single-point LOD scores of 1.3 and 0.55 were obtained in Branches 1 and 3, respectively, to chromosome 15q24.3-25 linkage region (see Table 4). The highest single-point LOD score of 2.26 revealed to rs11072823 when linkage analysis was performed in Branches 1 and 3 (keeping the ancestral relationship intact) and the highest multipoint LOD score increased to 2.65 at rs11072823 (see Table 5). Although the highest LOD scores are suggestive, the branch-wise analysis provides further support for linkage at 15q to SLI (see Supplemental Figure S2).

**Table 4.** Single-point LOD scores (15q region) in Family 315, branches, and possible combinations.

rsID	hg19 position (Mbp)	Family 315	Branch 1	Branch 2	Branch 3	Branches 1 and 2	Branches 1 and 3	Branches 2 and 3
rs1075991	68.68	−5.15	−0.67	−2.27	0.13	−3.08	−0.47	−2.32
rs896999	69.10	0.36	0.00	0.12	0.03	0.00	0.15	0.28
rs748681	69.20	−2.97	1.33	−4.40	0.04	0.59	1.74	−4.45
rs2415047	69.89	−1.32	0.72	−2.27	0.03	−0.95	0.88	−2.12
rs8035183	70.33	−3.90	0.43	−4.40	0.13	0.28	0.63	−4.36
rs2001597	70.42	−3.31	1.33	−5.10	0.04	−0.76	1.74	−4.94
rs652937	71.47	0.63	0.00	0.00	0.42	0.18	0.55	0.55
rs11632479	72.01	0.36	0.00	0.12	0.03	0.00	0.15	0.28
rs12594531	72.07	−3.44	0.72	−4.40	0.03	0.88	0.88	−4.24
rs2957740	72.31	−1.74	0.72	−2.70	0.03	0.89	0.88	−2.55
rs1374092	72.81	0.36	0.00	0.12	0.03	0.00	0.15	0.28
rs11632630	73.25	−1.39	0.72	−2.27	0.04	−1.81	0.84	−2.12
rs12903941	73.55	0.36	0.00	0.12	0.03	0.00	0.15	0.28
rs17773784	74.08	−2.17	0.00	−2.40	0.03	−1.23	0.15	−2.25
rs896588	74.11	−6.06	0.43	−7.10	0.55	0.28	1.36	−6.64
rs1823718	74.15	−5.67	1.33	−7.10	0.04	0.59	1.74	−7.15
rs1484214	74.58	−1.60	0.43	−2.27	0.13	−1.69	0.63	−2.32
rs741761	74.70	−3.77	0.00	−4.40	0.42	−0.01	0.55	−3.85
rs2075590	74.71	−2.17	0.00	−2.40	0.03	−1.23	0.15	−2.25
rs2075589	74.71	−2.17	0.00	−2.40	0.03	−1.23	0.15	−2.25
rs11857558	74.71	0.36	0.00	0.12	0.03	0.00	0.15	0.28
rs11852760	74.72	−1.65	0.00	−2.27	0.42	−1.40	0.55	−1.73
rs1992145	74.72	−1.65	0.00	−2.27	0.42	−1.40	0.55	−1.73
rs12898794	74.88	−4.87	0.00	−5.10	0.03	−1.40	0.15	−4.95
rs41279188	75.01	−4.04	0.00	−4.27	0.03	0.59	0.15	−4.12
rs762551	75.04	0.63	0.00	0.00	0.42	0.18	0.55	0.55
rs2470890	75.05	0.63	0.00	0.00	0.42	0.18	0.55	0.55
rs12050778	76.13	−4.21	0.43	−5.10	0.04	−1.89	0.84	−4.94
rs2955736	76.38	−1.77	0.00	−2.40	0.42	−1.23	0.55	−1.85
rs744336	76.67	−1.77	0.00	−2.40	0.42	−2.36	0.55	−1.85
rs11636648	77.34	−3.32	0.72	−4.27	0.03	1.48	0.88	−4.12
rs11639314	77.54	−4.04	0.00	−4.27	0.03	0.59	0.15	−4.12
rs74025333	77.93	−2.17	0.00	−2.40	0.03	−1.23	0.15	−2.25
rs4243047	77.98	0.63	0.00	0.00	0.42	−0.01	0.55	0.55
rs1519819	78.54	0.93	0.00	0.60	0.42	0.17	0.55	0.85
rs965604	78.79	0.36	0.00	0.12	0.03	0.00	0.15	0.28
**rs11072823**	**79.29**	**3.06**	**1.33**	**0.73**	**0.55**	**0.92**	**2.26**	**1.35**
rs1402760	79.44	−6.14	0.72	−7.10	0.03	0.88	0.88	−6.94
rs4778752	80.40	−2.47	0.00	−2.70	0.03	−2.36	0.15	−2.55
rs2034247	80.61	0.63	0.00	0.00	0.42	0.46	0.55	0.55
rs11072930	80.91	−1.81	0.43	−2.70	0.04	−0.01	0.84	−2.54
rs1553650	81.11	1.57	1.33	0.00	0.42	0.59	1.53	−0.05
rs4075641	81.78	1.08	0.72	0.12	0.03	0.89	0.88	0.28
rs1567897	82.02	−6.87	0.00	−7.10	0.03	−2.40	0.15	−6.94
rs12916134	82.19	−3.05	0.72	−4.40	0.42	−1.33	1.27	−3.85
rs1267657	83.22	−5.35	0.43	0.12	−4.70	−0.01	−4.51	−4.58

*Note.* The parameters were set for recessive inheritance, omnibus language affected, full penetrance, and a disease frequency of 0.001. The SNP in bold has the highest single-point LOD score when linkage analysis included all members of Family 315. Mbp = megabases.

**Table 5.** Highest multipoint LOD scores within chromosome 15q24.3-q25 in Family 315 branch analysis.

Analysis	Branches included	Multipoint LOD score
Individual branches	Branch 1	1.33
	Branch 2	−7.5
	Branch 3	0.73
One branch removed; other relationships intact	Branch 1 and 2	0.29
	Branches 1 and 3	2.65
	Branches 2 and 3	−7.12

*Note.* The parameters were set for recessive inheritance, omnibus language affected, full penetrance, and a disease frequency of 0.001.

## Discussion

In this study, we performed a parametric genome-wide scan in SLI families with multiple omnibus affected individuals. Our study suggested new linkage loci and confirmed previously reported loci in SLI, suggesting locus heterogeneity. We reported three independent linkage loci in three families that reach the likelihood of suggestive linkage (see Table 3). Though these results are suggestive, all three regions are uniquely important in their contribution to explaining transmission of SLI. In Family 300, a large suggestive region was identified under recessive inheritance and the family's inheritance pattern of performance on the omnibus measure shows a clear recessive pattern. In Family 489, the region identified includes a previously reported gene showing a paternal parent-of-origin effect, and the gene is involved in mechanisms implicated in other neurological disorders. Finally, the largest family (315) provides further support for family-based analysis and reveals a novel region of interest. In the other three families, suggestive linkage was not estimated, possibly due to family size or structure (see Figure 1).

Our findings are consistent with the conclusion that genome-wide parametric linkage analysis is an authentic and powerful method to map disease genes in disorders like SLI, dyslexia, and stuttering, given the phenotype is well characterized and mapped to multiple individuals in the family (Nopola-Hemmi et al., 2001; Raza et al., 2012 , 2013; Truong et al., 2016). The outcomes here are consistent with the use of family-based linkage mapping to provide sufficient information to identify DNA variations in genes responsible for stuttering, a speech phenotype (Raza et al., 2012 , 2013). With easy access to next NGS and its reduced cost over time, researchers adopt NGS to identify disease associated genes (Koboldt et al., 2013). We recognize that this is mostly useful and cost-effective for rare/Mendelian disorders but is less successful for non-Mendelian disorders. For complex phenotypes, like SLI, initial gene mapping information is likely to provide genomic targets to prioritize DNA variations in NGS data (Bailey-Wilson & Wilson, 2011; Hastings et al., 2016; Raza et al., 2013, 2015). The linkage data presented in this article, although suggestive, will be useful to narrow the focus for gene identification efforts through targeted/whole genome/exome sequencing.

Our study follows the precedents in the literature for phenotyping SLI using standard scores. However, previous methods differ from those used in this study in ways that may matter in sensitivity to detection of genetic effects. As noted earlier, the CELF-R (omnibus language measure) was used by the SLIC and linkage loci were reported (SLIC, 2002). Their analysis divided the CELF language phenotype into the two component subtests: Receptive and Expressive Language scores. Although the initial analyses revealed linkage with the CELF-R expressive language score on chromosome 19q, the follow-up analyses did not replicate these results (SLIC, 2002, 2004). In additional follow-up analyses by the SLIC, multivariate linkage analysis was used to simultaneously analyze multiple CELF-R subtests (Monaco & SLI Consortium, 2007). The multivariate analysis indicated the 16q locus may be involved in language, reading, and spelling phenotypes, as well as NWR performance, while the 19q locus was not strongly linked to reading and spelling (Monaco & SLI Consortium, 2007). These results indicate support for initial genetic analysis utilizing omnibus phenotypes; in that language, reading, spelling, and memory may share common causal pathways, especially given the prevalence of individuals with both language and reading difficulties. Other studies have used omnibus language measures in conjunction with additional language measures to assign affection status (Villanueva et al., 2010, 2011, 2008) or focused on performance on the NWR task, following up on the early results from the SLIC (Newbury et al., 2009; Whitehouse et al., 2011). A recent report showed high heritability of performance on omnibus language measures, in 6-year-old twins, using the TOLD-P:3, a more recent version of the TOLD measure used in this study (Newcomer & Hammill, 1997; Rice et al., 2020). In the context of previous studies, the study reported here contributes new information about omnibus language phenotypes, for the purpose of grouping participants as SLI. Further studies are needed to establish replicability of omnibus phenotypes. Replicability could be influenced due to the use of omnibus measures to establish affectedness status, as more recent research suggests. Perhaps more importantly and more likely, replicability could also be influenced by the level of standards in place for training examiners and possible differences across studies in the editions of tests carrying the same name.

Omnibus measures are also of interest as a phenotype because of the possible insight they can provide into the overlap with other related disorders that may share common causal pathways. For example, recall that early language impairments predict later reading impairments. Specifically, evidence from a longitudinal study of children with SLI shows that those with the poorest reading comprehension skills had significantly lower scores on both the Peabody Picture Vocabulary Test–Fourth Edition and the CELF in eighth grade, as compared to those who had the poorest reading decoding skills (Catts et al., 2006). These results show that while reading involves both decoding and language knowledge, underlying language deficits play a more crucial role in overall comprehension of text (Catts et al., 2006). These behavioral and genetic results combined leave an open question in terms of the potential of genetic study of related phenotypes. Three regions previously reported in RD, chromosome 1p36, 3p12, and 18p11, overlapped with regions where we observed LOD scores above 1.0 in Families 315, 387, and 430 (see Supplemental Table S1; Fisher & Francks, 2006; Fisher et al., 2002; Grigorenko et al., 2001; Hannula-Jouppi et al., 2005; Nopola-Hemmi et al., 2001; Rabin et al., 1993; Tzenova et al., 2004). This overlap also supports the fact that the participants in the current study were included in the previous targeted investigation of reported RD loci, including 1p36, 3p12-q13.

In the context of the many candidate SLI regions and genes already reported, only one candidate gene *NOP9* overlapped with our three reported suggestive regions, though other regions with LOD scores greater than 1.2 in four families (315, 387, 430, and 489) are also noted if they coincided with previously reported regions or candidate genes associated with SLI, RD, NWR performance, ASD, and CAS (see Supplemental Table S1; Chen et al., 2017; Mountford & Newbury, 2018; Mountford et al., 2019). A few candidate genes of note overlapped with the regions showing markers with LOD scores above 1.2 including *ROBO1*, *ROBO2*, and *FOXP1* (see Supplemental Table S1; Chen et al., 2017; Mountford & Newbury, 2018; Mountford et al., 2019). The lack of overlap of the reported region and some of the most promising candidate genes, like *TM4SF20*, *NFXL1*, and *SETBP1*, is not surprising given that these were identified in unique populations or appear to be linked to language in a specific race, in the case of *TM4SF20* (Rakhlin et al., 2013; Villanueva et al., 2011; Wiszniewski et al., 2013). The previous results emphasized focused investigations of well-characterized samples with similar genetic backgrounds in the identification of causative genes. Our sample and procedures are consistent with these criteria, supporting the utility of the current foundational study.

Other challenges for gene discovery are at the level of variations in the size of gene regions of interest. The linkage region (~25 MB) in Family 300 is large, which could be due to a small family size. However, this could be a new SLI locus to be considered in the future with a larger data set. This locus may be a useful target for genome sequencing in this family showing a recessive inheritance patterns for language phenotypes. Additionally, some SNPs at 4q with the LOD score above 1.2 were observed in Families 387 and 430, indicating replication, although with less significance (see Supplemental Table S1). This overlap could help to narrow the region of interest in follow-up investigation of exome variations.

Another feature of interest with Family 300 is that previous reports overlapping with the 4q region include a case study of ASD in which a deletion was identified on 4q (Ramanathan et al., 2004). The one child with ASD in our sample is in Family 300. Therefore, we conducted follow-up analysis excluding the child with ASD, revealing that the LOD score on chromosome 4q decreased to 1.8, while LOD scores in two other regions increased to 1.8 (chr7p14.3-p22.2 and chr22q11.21-q12.3). Notably, the chromosome 7p and 22q regions did not contain any markers with negative LOD scores. The chromosome 22q region overlaps with two previous reports (Gialluisi et al., 2014; Landi et al., 2013). One end of the 22q region overlaps with an SNP in *RBFOX2* gene (RNA binding Fox-1 homolog 2) associated with language and reading phenotypes (Gialluisi et al., 2014). The other end of the boundary overlaps with the gene *COMT,* in which a variation has been associated with better readers (Landi et al., 2013). Although these regions are consistent with the previous reports, the linkage findings in Family 300 are not definitive, warranting further investigation.

The overlapping report of the suggestive region we report on chromosome 14 in Family 489 (14q11.2-q13.3) is especially noteworthy. A paternal parent-of-origin effect was identified previously at chromosome 14q12, specifically a nonsynonymous variant in the *NOP9* gene (nucleolar protein 9; Nudel et al., 2014; Pettigrew et al., 2016). Note that, in Family 489, the father is the affected parent (see Figure 1). However, we did not see an indication of paternal parent-of-origin effect based on our analysis of the pattern of inheritance in this region. The SNP closest to the *NOP9* gene available on our array is rs1957528, and its LOD score is equal to the highest observed LOD score of 2.4 in Family 489 (see Table 2). The NOP9 protein is linked to RNA-binding proteins, which have been associated with multiple neurological disorders (Kapeli & Yeo, 2012; Nudel et al., 2014). *NOP9* encodes a nucleolar protein that is involved in processing ribosomal RNA. Cells depleted with NOP9 were unable to process the mature ribosomal RNA, which is an important component of ribosome assembly (Thomson et al., 2007). Efficient ribosomal assembly is crucial for protein synthesis and is involved in chromatin organization. There is growing evidence that dysregulated ribosomal biogenesis results in neurodevelopmental disorders like autism, intellectual disability, and progressive neurodegenerative disorders (Hetman & Slomnicki, 2019). It is also worth mentioning that the 14q region overlaps with our previously reported homozygosity region in consanguineous families with language deficits, from 14q12-q32.2 (Andres et al., 2019). Other genes within the 14q region may also be considered strong candidates because this region reported in multiple studies previously.

**Figure 2.**
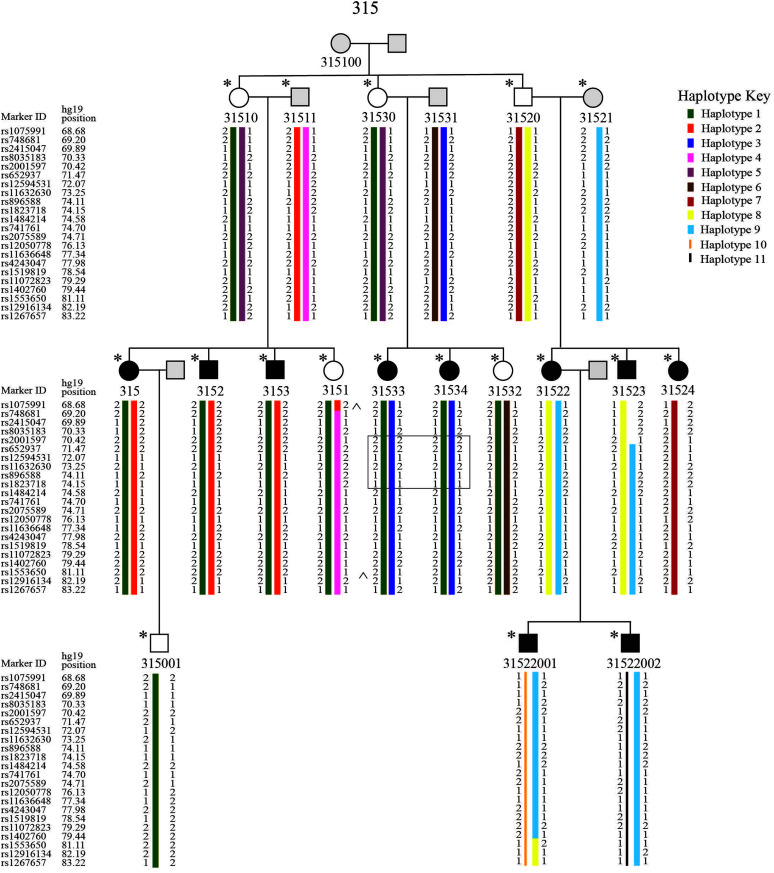
Haplotype assignments in Family 315 at chromosome 15q23-q25.2 (68.68–83.22 Mb). ^ indicates the cross-over events in 3151 and 31533, * indicates the genotyped individuals, and the solid outline indicates a homozygous region in Individuals 31533 and 31534.

We noted in the introduction that one of the advantages of pedigree analysis is that it can reduce trait variance of complex phenotypes at the behavioral level in order to better capture the genetic factors involved in the transmission of the trait (Neale & Cardon, 2013). Yet, additional factors must be considered, at more detailed levels of analysis. For example, inclusion of additional extended family members may introduce phenotypic and genotypic variance that must be parsed out. In the case of Family 315, the largest family in the study, additional branch-wise and branch combination analysis further supported and narrowed the region of interest. The branch-wise analysis revealed that Branch 2 contributed to the reduced calculated LOD score in Family 315 compared to the ELOD. This could mean the language phenotype within Branch 2 varies more meaningfully in comparison to the members of the other branches. To understand how the alleles are inherited to the 15q locus in Family 315, we computed haplotypes, using MERLIN and Haplopainter (see Figure 2; Abecasis et al., 2002; Thiele & Nürnberg, 2004). Two cross-over events delimit the 14.54-Mb linkage region in Branches 1 and 3 (see ^ on Figure 2). Two haplotypes (Haplotypes 1 and 2) cosegregate in Branch 1 and a similar pattern observed in Branch 3 but with Haplotypes 1 and 3 (see Figure 2). We observed that an affected individual, 31524, in Branch 2, inherited Haplotype 1, but this haplotype and Haplotype 2 or 3 were not identified in other affected individuals of Branch 2, indicating that there might be another locus segregating in this branch of the family. Haplotype 1 is the ancestral haplotype shared among all affected in Branches 1 and 3. Haplotypes 2 and 3 are inherited from the married-in individuals and co-occur with Haplotype 1 in the affected members of Branches 1 and 3, indicating that more than one haplotype segregates with the trait in Branches 1 and 3. We also noticed that Haplotype 2 and Haplotype 3 shared a small section (74.15-77.34 Mb; on Figure 2), which indicates the possibility that 31511 and 31531 are distantly related or an unknown ancestral recombination occurred. Also, affected members of Branch 1 share a ~3-Mb region of homozygosity (77.98–81.11 Mb; on Figure 2) containing the highest scoring marker. A founder effect is suggested when multiple haplotypes or copies of the same haplotype are segregated in the pedigree by an unknown distant founder (Drayna, 2005). Despite the high scoring markers under the recessive model of inheritance, Family 315 did not show loss of heterozygosity regions greater than 4 Mb, the size that is reportedly common in outbred populations (McQuillan et al., 2008). Additionally, inspection of the region shared by affected members of Branch 1 (77.98–81.11 Mb) showed one heterozygous marker and homozygosity analysis of Branches 1 and 3 (relationships intact, Branch 2 removed) did not reveal any homozygosity regions. However, homozygosity analysis of Branches 1 and 3 individually showed homozygous regions between 3.5 and 4 Mb on chromosome 15q in affected members (chr15:47686263-51204364 in Branch 1 and chr15:70421861-74147244 in Branch 3), though only the homozygous region in Branch 3 (solid outline on Figure 2) overlapped with the reported suggestive linkage region. Interestingly, the homozygous region in the affected members of Branch 3 indicates Haplotypes 1 and 3 could be part of the same ancestral chromosome as it appears in individual 31531, though the genotypes are inferred. Additionally, one homozygous region was shared by affected individuals of Branch 1 on chromosome 14q (chr14:333909916-37059525), but it was still less than 4 Mb. The linkage results on chromosome 14q in Branch 1 shows positive LOD scores, but no markers showing LOD scores greater than the highest background LOD score in Branch 1 (see Supplemental Figure S2). Unlike with the region on chromosome 15q, no overlap was observed with other branches individually or in combination, indicating the homozygous region on 14q may not be of further interest in Family 315. Our haplotype analysis suggested a possibility of a distant common ancestor, but genome-wide homozygosity analysis negates it. The segregation of more than one haplotype in Branches 1 and 3 on chromosome 15q indicates that multiple alleles at this locus may be the cause of SLI in Family 315.

The SNP on chromosome 15, rs11072823 that reached an LOD score of 3.06, is also of interest because it is found on *RasGRF1* (Ras protein specific guanine nucleotide releasing factor). RasGRF1 is a regulator of the extracellular signal-regulated kinase signaling pathway via binding with a N-methyl-D-aspartate receptor subunit, specifically NR2B (Krapivinsky et al., 2003). RasGRF1 is directly activated by calcium and is exclusively expressed in the nervous system (Krapivinsky et al., 2003). Previous studies have disrupted the function of the RasGRF1 protein in mice and found effects on the learning and memory mechanisms controlled by the amygdala and the hippocampus (Brambilla et al., 1997; Giese et al., 2001). This could be another candidate for future investigation in Family 315 and other cases of SLI, due to the role this protein plays in learning and memory pathways.

## Conclusions

Overall, our family-based genetic study provides important genomic targets suggesting genotype–phenotype associations. We provided evidence of new loci and confirmed a previously reported locus. The replication of a previously reported SLI locus in the current study illustrates the strength of traditional family-based gene mapping. Our findings are consistent with the hypothesis that there are several loci involved in the transmission of SLI. Future family-based genetic studies and investigation of the reported loci are warranted. This is the first genome-wide linkage analysis of families coming from the longitudinal study of the KU cohort, providing an important foundational step for future identification of causal genetic markers involved in the regulation of gene pathways in individuals with SLI.

Although the results are encouraging, we recognize the limitations of a family-based study design. In particular, the design assumes that major gene effects are shared even though this is a complex disorder. It is also true that the replication across families brings more confidence in potential shared genetic effects across families. It remains for future studies to provide further clarification of the exact nature of gene effects.

Ultimately, replicated findings of specific genetic pathways may be helpful in clinical counseling with the families of children with SLI. Furthermore, it could increase the likelihood of identification of affected family members. Clinical speech/language pathology services could expand to a familial approach with support for the possible social and academic consequences of SLI, as well as the identification of clinical speech and language goals from phenotypes demonstrated to be associated with genetic influence, benchmarked to age expectations.

## Author Contributions

**Erin M. Andres:** Formal analysis (Lead), Investigation (Equal), Methodology (Equal), Writing – original draft (Equal), Writing – review & editing (Equal). **Kathleen Kelsey Earnest:** Data curation (Equal), Supervision (Equal), Writing – review & editing (Equal). **Shelley D. Smith:** Data curation (Equal), Writing – review & editing (Equal). **Mabel L. Rice:** Conceptualization (Equal), Funding acquisition (Lead), Project administration (Equal), Resources (Equal), Supervision (Lead), Writing – review & editing (Equal). **Muhammad Hashim Raza:** Conceptualization (Equal), Investigation (Equal), Methodology (Lead), Project administration (Equal), Resources (Equal), Supervision (Lead), Visualization (Lead), Writing – original draft (Equal), Writing – review & editing (Equal).

## Supplementary Material

10.1044/2020_JSLHR-20-00102SMS1 Supplemental Figure S1 Genome-wide linkage results for all six families.

10.1044/2020_JSLHR-20-00102SMS2 Supplemental Figure S2 Linkage analysis separately in Branch 1, Branch 2, and Branch 3 and all possible combinations of these branches.

10.1044/2020_JSLHR-20-00102SMS3 Supplemental Table S1 Previously reported chromosomal loci linked to related phenotypes showing single point logarithm of odds (LOD) scores > 1.2.

10.1044/2020_JSLHR-20-00102SMS4 Supplemental Table S2 Genes in the suggestive linkage region chr4:154,887,604-179,989,221 (chr4q31.3-q34.3).

10.1044/2020_JSLHR-20-00102SMS5 Supplemental Table S3 Genes in the suggestive linkage region chr4:154,887,604-179,989,221 (chr4q31.3-q34.3).

10.1044/2020_JSLHR-20-00102SMS6 Supplemental Table S4 Genes in the linkage region chr14:20,825,965-37,059,525 (chr14q11.2-q13.3).

10.1044/2020_JSLHR-20-00102SMS7 Supplemental Table S5 Additional single nucleotide polymorphisms (SNPs) with single-point logarithm of odds (LOD) scores > 2.0 in family 315 genome-wide.
