# Supplementary figures and images for: Pedigree-Based Gene Mapping Supports Previous Loci and Reveals Novel Suggestive Loci in Specific Language Impairment
Source: J Speech Lang Hear Res. 2020 Nov 13;63(12):4046–61. doi: 10.1044/2020_JSLHR-20-00102 (PMC8608229; doi:10.1044/2020_JSLHR-20-00102)

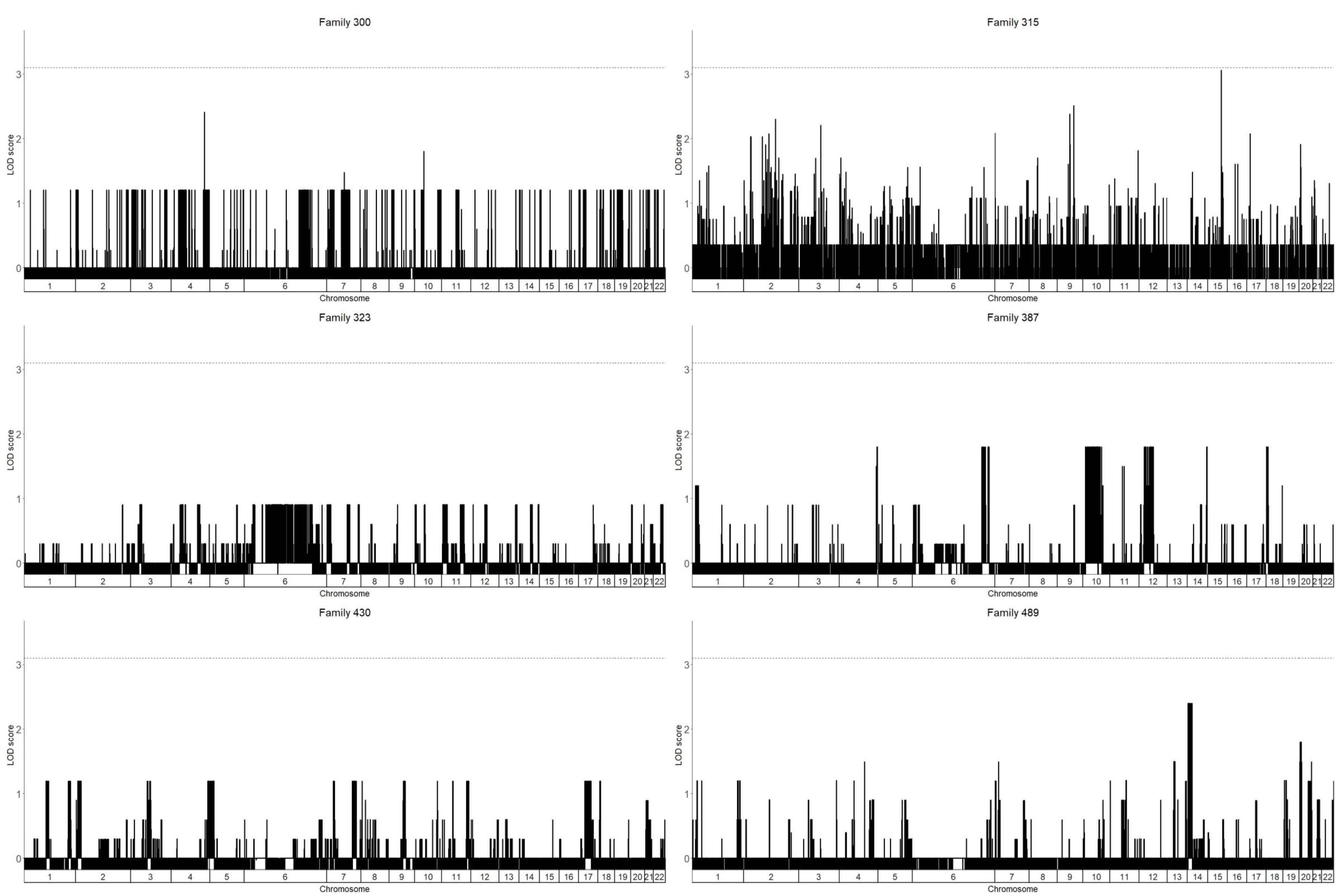

Supplement: Supplemental Figure S1 [file JSLHR-63-4046-s001.jpg]

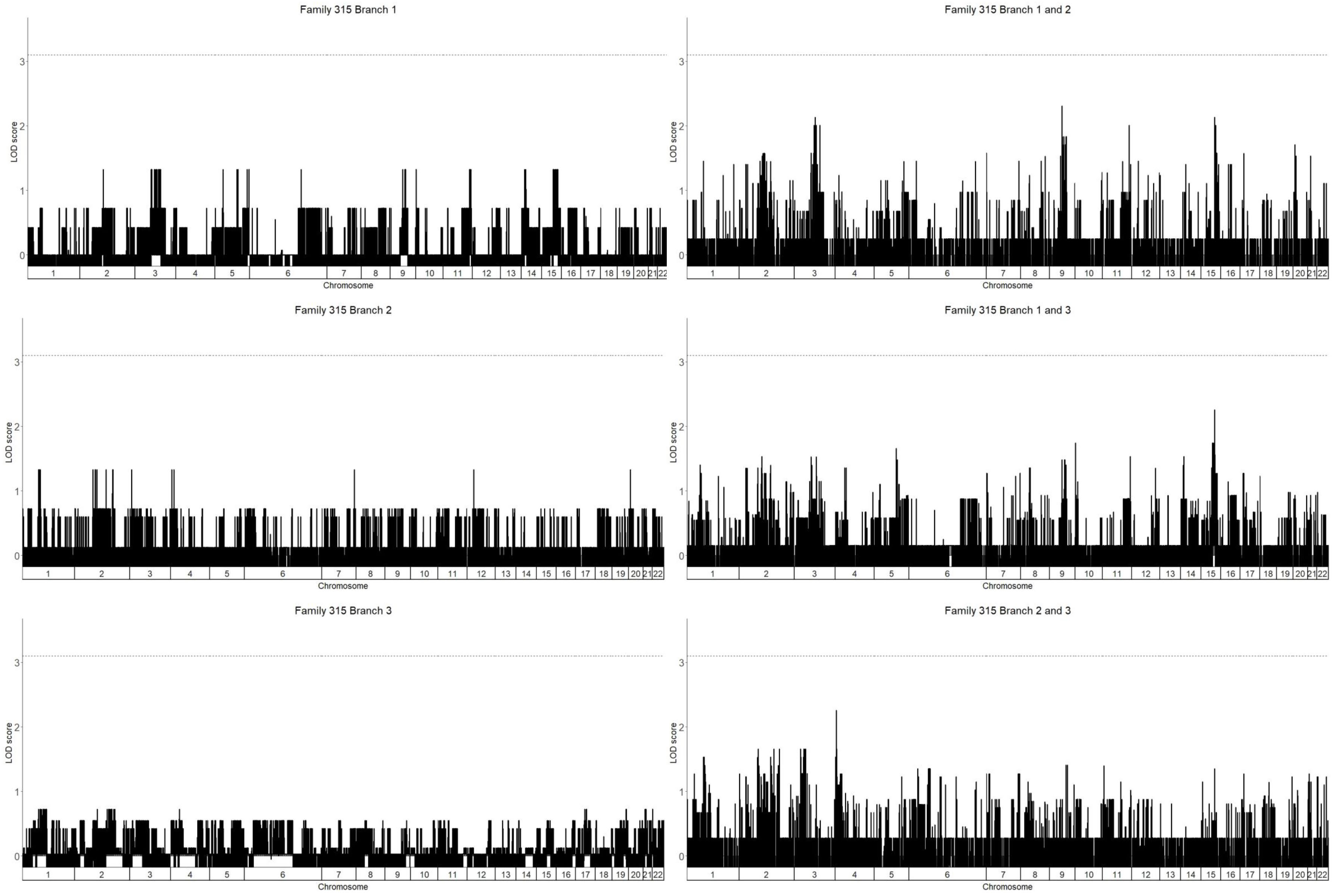

Supplement: Supplemental Figure S2 [file JSLHR-63-4046-s002.jpg]
