# Supplementary material for: Pedigree-Based Gene Mapping Supports Previous Loci and Reveals Novel Suggestive Loci in Specific Language Impairment
Source: J Speech Lang Hear Res. 2020 Nov 13;63(12):4046–61. doi: 10.1044/2020_JSLHR-20-00102 (PMC8608229; doi:10.1044/2020_JSLHR-20-00102)
Supplement: Supplemental Table S1 [file JSLHR-63-4046-s003.pdf]

Supplemental Table S1. Previously reported chromosomal loci linked to related phenotypes showing single point logarithm of odds (LOD) scores > 1.2.

Chr	Locus	hg19 Position (Mbp)	rsIDs (Start-End)	Family	Single-Point LOD score	Mode of Inheritance	Previously Linked/ Associated Phenotype	Reference
1	1p36.11-p36.12	22.62-24.40	rs6426746-rs4649175	315	1.45	Dominant	RD, SLI	(Grigorenko et al., 2001; Rabin et al., 1993; Rice et al., 2009; Tzenova et al., 2004)
	1p36.12-p36.23	8.61-23.73	rs1473420-rs3889814	387	1.80*	Dominant		
3	3p12.2-p13	71.52-83.38	rs17108-rs349148	387	1.80*	Dominant	RD, SLI ASD, CAS	(Chen et al., 2017; Fisher & Francks, 2006; Hannula-Jouppi et al., 2005; Mountford et al., 2019; Nopola-Hemmi et al., 2001; Rice et al., 2009; Smith, 2007)
	3p12.2-q13.11	82.21-105.80	rs17114-rs2035254	430	1.20*	Recessive		
4	4q35.1-q35.2	185.95-190.78	rs1564986-rs1915852	387	1.80*	Recessive	ASD	(Ramanathan et al., 2004; Smith, 2007)
	4q34.3-q35.2	180.48-190.31	rs2610998-rs1456351	430	1.20*	Recessive		
9	9q22.32-q33.1	98.44-117.82	rs1335049-rs1060545	430	1.20*	Recessive	CAS	(Laffin et al., 2012)
12	12p12.3-q21.1	16.06-73.44	rs10846247-rs1400142	387	1.80*	Recessive	NWR test, CAS	(Addis et al., 2010)
13	13q14.12-q14.3	45.63-52.54	rs1747109-rs1801244	489	1.50	Recessive	NWR test	(Truong et al., 2016)
18	18p11.23-p11.32	0.15-7.32	rs545684-rs569629	387	1.80*	Recessive	CAS, RD	(Fisher et al., 2002)
	18p11.21-p11.31	5.61-10.97	rs948311-rs264234	430	1.20*	Recessive		

Note. Chr = chromosome; Mbp = megabases; RD = reading disorder; SLI = specific language impairment; ASD = autism spectrum disorders; CAS = childhood apraxia of speech; NWR = nonword repetition. *LOD scores that reached the maximum estimated LOD (ELOD).

References

Addis, L., Friederici, A. D., Kotz, S. A., Sabisch, B., Barry, J., Richter, N., Ludwig, A. A., Rübsamen, R., Albert, F. W., Pääbo, S., Newbury, D. F., & Monaco, A. P. (2010). A locus for an auditory processing deficit and language impairment in an extended pedigree maps to 12p13.31-q14.3. *Genes, Brain and Behavior*, *9*(6), 545-561. https://doi.org/10.1111/j.1601-183X.2010.00583.x

Chen, X. S., Reader, R. H., Hoischen, A., Veltman, J. A., Simpson, N. H., Francks, C., Newbury, D. F., & Fisher, S. E. (2017). Next-generation DNA sequencing identifies novel gene variants and pathways involved in specific language impairment. *Scientific Reports*, *7*, 1-17, Article 46105. https://doi.org/10.1038/srep46105

Fisher, S. E., & Francks, C. (2006). Genes, cognition and dyslexia: Learning to read the genome. *Trends in Cognitive Sciences*, *10*(6), 250-257. https://doi.org/10.1016/j.tics.2006.04.003

Fisher, S. E., Francks, C., Marlow, A. J., MacPhie, I. L., Newbury, D. F., Cardon, L. R., Ishikawa-Brush, Y., Richardson, A. J., Talcott, J. B., Gayan, J., Olson, R. K., Pennington, B. F., Smith, S. D., DeFries, J. C., Stein, J. F., & Monaco, A. P. (2002). Independent genome-wide scans identify a chromosome 18 quantitative-trait locus influencing dyslexia. *Nat Genet*, *30*(1), 86-91. https://doi.org/10.1038/ng792

Grigorenko, E. L., Wood, F. B., Meyer, M. S., Pauls, J. E. D., Hart, L. A., & Pauls, D. L. (2001). Linkage studies suggest a possible locus for developmental dyslexia on chromosome 1p. *American Journal of Medical Genetics*, *105*(1), 120-129. https://doi.org/10.1002/1096-8628(20010108)105:1<120::AID-AJMG1075>3.0.CO;2-T

Hannula-Jouppi, K., Kaminen-Ahola, N., Taipale, M., Eklund, R., Nopola-Hemmi, J., Kääriäinen, H., & Kere, J. (2005). The axon guidance receptor gene *ROBO1* is a candidate gene for developmental dyslexia. *PLoS Genetics*, *1*(4), 0467-0474, Article e50. https://doi.org/10.1371/journal.pgen.0010050

Laffin, J. J. S., Raca, G., Jackson, C. A., Strand, E. A., Jakielski, K. J., & Shriberg, L. D. (2012). Novel candidate genes and regions for childhood apraxia of speech identified by array comparative genomic hybridization. *Genetics in Medicine*, *14*(11), 928-936. https://doi.org/10.1038/gim.2012.72

Mountford, H. S., Villanueva, P., Fernández, M. A., De Barbieri, Z., Cazier, J. B., & Newbury, D. F. (2019). Candidate gene variant effects on language disorders in Robinson Crusoe Island. *Annals of Human Biology*, *46*(2), 109-119. https://doi.org/10.1080/03014460.2019.1622776

Nopola-Hemmi, J., Myllyluoma, B., Haltia, T., Taipale, M., Ollikainen, V., Ahonen, T., Voutilainen, A., Kere, J., & Widén, E. (2001). A dominant gene for developmental dyslexia on chromosome 3. *Journal of Medical Genetics*, *38*(10), 658-664. https://doi.org/10.1136/jmg.38.10.658

Rabin, M., Wen, X. L., Hepburn, M., Lubs, H. A., Feldman, E., & Duara, R. (1993). Suggestive linkage of developmental dyslexia to chromosome 1p34-p36. *The Lancet*, *342*(8864), 178. https://doi.org/10.1016/0140-6736(93)91384-X

Ramanathan, S., Woodroffe, A., Flodman, P. L., Mays, L. Z., Hanouni, M., Modahl, C. B., Steinberg-Epstein, R., Bocian, M. E., Spence, M. A., & Smith, M. (2004). A case of autism with an interstitial deletion on 4q leading to hemizygosity for genes encoding for glutamine and glycine neurotransmitter receptor sub-units (*AMPA 2*, *GLRA3*, *GLRB*) and neuropeptide receptors *NPY1R*, *NPY5R*. *BMC Medical Genetics*, *5*(1), Article 10. https://doi.org/10.1186/1471-2350-5-10

Rice, M. L., Smith, S. D., & Gayán, J. (2009). Convergent genetic linkage and associations to language, speech and reading measures in families of probands with specific language impairment. *Journal of Neurodevelopmental Disorders*, *1*(4), 264-282. https://doi.org/10.1007/s11689-009-9031-x

Smith, S. D. (2007). Genes, language development, and language disorders. *Mental Retardation and Developmental Disabilities Research Reviews*, *13*(1), 96-105. https://doi.org/10.1002/mrdd.20135

Truong, D. T., Shriberg, L. D., Smith, S. D., Chapman, K. L., Scheer-Cohen, A. R., DeMille, M. M. C., Adams, A. K., Nato, A. Q., Wijsman, E. M., Eicher, J. D., & Gruen, J. R. (2016). Multipoint genome-wide linkage scan for nonword repetition in a multigenerational family further supports chromosome 13q as a locus for verbal trait disorders. *Human Genetics*, *135*(12), 1329-1341. https://doi.org/10.1007/s00439-016-1717-z

Tzenova, J., Kaplan, B. J., Petryshen, T. L., & Field, L. L. (2004). Confirmation of a dyslexia susceptibility locus on chromosome 1p34-p36 in a set of 100 Canadian families. *American Journal of Medical Genetics Part B: Neuropsychiatric Genetics*, *127*(1), 117-124. https://doi.org/10.1002/ajmg.b.20139
