# Supplementary material for: Pedigree-Based Gene Mapping Supports Previous Loci and Reveals Novel Suggestive Loci in Specific Language Impairment
Source: J Speech Lang Hear Res. 2020 Nov 13;63(12):4046–61. doi: 10.1044/2020_JSLHR-20-00102 (PMC8608229; doi:10.1044/2020_JSLHR-20-00102)
Supplement: Supplemental Table S2 [file JSLHR-63-4046-s004.pdf]

Supplemental Table S2. Genes in the suggestive linkage region chr4:154,887,604-179,989,221 (chr4q31.3-q34.3).

DCHS2	RXFP1	TRIM61	LINC02275	CEP44
PLRG1	C4orf46	FAM218A	MFAP3L	MIR4276
FGB	ETFDH	TRIM60	AADAT	HPGD
FGA	PPID	TMEM192	LINC01612	GLRA3
FGG	FNIP2	KLHL2	LINC02382	LOC101928551
LRAT	C4orf45	GK3P	LINC02431	ADAM29
RBM46	RAPGEF2	MSMO1	MIR6082	GPM6A
NPY2R	MIR3688-1	CPE	LINC02174	LOC101928590
MAP9	MIR3688-2	MIR578	LOC441052	WDR17
LOC102724776	LINC02233	LINC01179	GALNTL6	SPATA4
GUCY1A1	FSTL5	LOC101928131	GALNTL6-AS1	ASB5
GUCY1B1	LOC101928052	TLL1	LOC101930370	SPCS3
ASIC5	MIR4454	SPOCK3	GALNT7	VEGFC
TDO2	NAF1	ANXA10	MIR548T	LINC02509
CTSO	NPY1R	DDX60	HMGB2	NEIL3
PDGFC	NPY5R	DDX60L	SAP30	AGA
GLRB	TKTL2	PALLD	SCRG1	LINC01098
GRIA2	TMA16	CBR4	HAND2	LINC01099
LINC02433	MARCHF1	SH3RF1	HAND2-AS1	
GASK1B	ANP32C	NEK1	LINC02269	
FAM198B-AS1	SMIM31	CLCN3	LINC02268	
TMEM144	APELA	HPF1	FBXO8	
