# Supplementary material for: Pedigree-Based Gene Mapping Supports Previous Loci and Reveals Novel Suggestive Loci in Specific Language Impairment
Source: J Speech Lang Hear Res. 2020 Nov 13;63(12):4046–61. doi: 10.1044/2020_JSLHR-20-00102 (PMC8608229; doi:10.1044/2020_JSLHR-20-00102)
Supplement: Supplemental Table S3 [file JSLHR-63-4046-s005.pdf]

Supplemental Table S3. Genes in the suggestive linkage region chr15:68,679,181-83,215,251 (chr15q23-q25.2).

ITGA11	ARIH1	CYP1A2	MIR3713	ST20-MTHFS
CORO2B	MIR630	CSK	RCN2	ST20
ANP32A	LINC02259	MIR4513	PSTPIP1	ST20-AS1
MIR4312	GOLGA6B	LMAN1L	TSPAN3	BCL2A1
ANP32A-IT1	HIGD2B	CPLX3	PEAK1	ZFAND6
NOX5	BBS4	ULK3	LINC00597	FAH
SPESP1	ADPGK	MIR6882	HMG20A	CTXND1
EWSAT1	ADPGK-AS1	SCAMP2	LOC101929457	LINC00927
GLCE	NEO1	MPI	LINGO1	ARNT2
PAQR5	MIR12135	FAM219B	LINGO1-AS1	LOC101929586
LOC145694	HCN4	COX5A	LINGO1-AS2	MIR5572
KIF23	REC114	RPP25	LOC645752	ABHD17C
RPLP1	NPTN	SCAMP5	COMMD4P1	CEMIP
DRAIC	NPTN-IT1	PPCDC	LOC91450	MIR549A
PCAT29	CD276	C15orf39	TBC1D2B	MESD
LINC00593	INSYN1	LOC105376731	SH2D7	MIR4514
TLE3	INSYN1-AS1	GOLGA6C	CIB2	TLNRD1
MIR629	TBC1D21	GOLGA6D	IDH3A	CFAP161
LINC02205	LOXL1-AS1	COMMD4	ACSBG1	IL16
LINC02204	LOXL1	NEIL1	DNAJA4	STARD5
SALRNA3	STOML1	MIR631	WDR61	TMC3-AS1
SALRNA2	PML	MAN2C1	CRABP1	TMC3
UACA	GOLGA6A	SIN3A	IREB2	MEX3B
LARP6	COMMD4P2	PTPN9	HYKK	LINC01583
LRRC49	LOC283731	SNUPN	PSMA4	EFL1
THAP10	ISLR2	IMP3	CHRNA5	SAXO2
THSD4	ISLR	SNX33	CHRNA3	ADAMTS7P1
THSD4-AS1	STRA6	CSPG4	CHRNB4	GOLGA6L10
THSD4-AS2	CCDC33	ODF3L1	LOC646938	UBE2Q2P2
NR2E3	CYP11A1	DNM1P35	ADAMTS7	GOLGA6L9
MYO9A	PPIAP46	MIR4313	MORF4L1	GOLGA2P10
SENP8	LINC02255	UBE2Q2	CTSH	LOC727751
GRAMD2A	SEMA7A	FBXO22	RASGRF1	GOLGA6L17P
PKM	MIR6881	NRG4	LOC100129540	RPS17
PARP6	UBL7	TMEM266	ANKRD34C-AS1	LOC102724034
CELF6	UBL7-AS1	LOC101929439	MIR184	CPEB1
HEXA	ARID3B	ETFA	ANKRD34C	
HEXA-AS1	CLK3	TYRO3P	TMED3	
TMEM202	EDC3	ISL2	MINAR1	
TMEM202-AS1	CYP1A1	SCAPER	MTHFS	
