# Supplementary material for: Pedigree-Based Gene Mapping Supports Previous Loci and Reveals Novel Suggestive Loci in Specific Language Impairment
Source: J Speech Lang Hear Res. 2020 Nov 13;63(12):4046–61. doi: 10.1044/2020_JSLHR-20-00102 (PMC8608229; doi:10.1044/2020_JSLHR-20-00102)
Supplement: Supplemental Table S4 [file JSLHR-63-4046-s006.pdf]

Supplemental material, Andres et al., "Pedigree-Based Gene Mapping Supports Previous Loci and Reveals Novel Suggestive Loci in Specific Language Impairment," *JSLHR*, https://doi.org/10.1044/2020_JSLHR-20-00102

Supplemental Table S4. Genes in the linkage region chr14:20,825,965-37,059,525 (chr14q11.2-q13.3).

PARP2	OR10G3	NGDN	SDR39U1	EGLN3
TEP1	OR10G2	THTPA	LOC101927045	SPTSSA
KLHL33	OR4E2	ZFHX2	CMA1	EAPP
OSGEP	OR4E1	AP1G2	CTSG	RNVU1-18
APEX1	LOC105370401	LOC102724814	GZMH	RNU1-3
PIP4P1	LINC02332	JPH4	GZMB	RNU1-4
PNP	DAD1	DHRS2	STXBP6	RNU1-2
RNASE10	ABHD4	DHRS4-AS1	NOVA1	RNU1-1
RNASE9	OR6J1	DHRS4	LINC02588	SNX6
RNASE11	OXA1L	DHRS4L2	LINC02294	CFL2
LOC254028	SLC7A7	DHRS4L1	LINC02293	BAZ1A
RNASE12	MRPL52	CARMIL3	MIR4307HG	LOC112268124
OR6S1	MMP14	CPNE6	MIR4307	SRP54-AS1
ANG	LRP10	NRL	LOC728755	IGBP1P1
RNASE4	REM2	PCK2	LINC00645	SRP54
EDDM3A	RBM23	DCAF11	MIR3171	FAM177A1
EDDM3B	PRMT5-AS1	FITM1	FOXG1-AS1	LOC101927178
RNASE6	PRMT5	PSME1	FOXG1	PPP2R3C
RNASE1	LOC101926933	EMC9	LINC01551	PRORP
RNASE3	HAUS4	PSME2	LINC02282	PSMA6
ECRP	MIR4707	MIR7703	PRKD1	NFKBIA
RNASE2	AJUBA	RNF31	G2E3-AS1	INSM2
METTL17	C14orf93	IRF9	G2E3	RALGAPA1
LOC101929718	PSMB5	REC8	SCFD1	SNORA101B
SLC39A2	PSMB11	IPO4	COCH	BRMS1L
NDRG2	CDH24	TM9SF1	LOC100506071	LINC00609
MIR6717	ACIN1	TSSK4	STRN3	PTCSC3
TPPP2	C14orf119	CHMP4A	MIR624	MBIP
RNASE13	LMLN2	MDP1	AP4S1	SFTA3
RNASE7	CEBPE	NEDD8-MDP1	HECTD1	NKX2-1
RNASE8	SLC7A8	NEDD8	HEATR5A	NKX2-1-AS1
ARHGEF40	RNF212B	GMPR2	LOC101927124	NKX2-8
ZNF219	HOMEZ	TINF2	DTD2	
TMEM253	PPP1R3E	TGM1	GPR33	
OR5AU1	BCL2L2-PABPN1	RABGGTA	NUBPL	
LINC00641	BCL2L2	DHRS1	LINC02313	
HNRNPC	PABPN1	NOP9	ARHGAP5-AS1	
RPGRIP1	SLC22A17	CIDEB	ARHGAP5	
SUPT16H	EFS	LTB4R2	RNU6-1	
CHD8	IL25	LTB4R	RNU6-9	
SNORD9	CMTM5	ADCY4	RNU6-2	
SNORD8	MYH6	RIPK3	RNU6-7	
RAB2B	MIR208A	NFATC4	RNU6-8	
TOX4	MYH7	NYNRIN	AKAP6	
METTL3	MHRT	CBLN3	NPAS3	
SALL2	MIR208B	KHNYN	SNORA89	
