# Supplementary material for: Pedigree-Based Gene Mapping Supports Previous Loci and Reveals Novel Suggestive Loci in Specific Language Impairment
Source: J Speech Lang Hear Res. 2020 Nov 13;63(12):4046–61. doi: 10.1044/2020_JSLHR-20-00102 (PMC8608229; doi:10.1044/2020_JSLHR-20-00102)
Supplement: Supplemental Table S5 [file JSLHR-63-4046-s007.pdf]

Supplemental Table S5. Additional single nucleotide polymorphisms (SNPs) with single-point logarithm of odds (LOD) scores > 2.0 in family 315 genome-wide.

SNPs	Chr	hg19 Position (Mbp)	Single-Point LOD score
rs2053372	2	24.91	2.03
rs935172	2	26.80	2.03
rs1386382	2	78.02	2.03
rs1545133	2	113.31	2.08
rs934748	2	113.36	2.08
rs1907642	3	111.74	2.21
rs7785205	7	0.93	2.08
rs2780701	9	93.57	2.38
rs984071	9	113.14	2.51
rs12450112	17	12.21	2.08

Note. Chr = chromosome; Mbp = megabases.
